# Clinical trials on 3D bioprinted scaffolds for lower extremity vascular, diabetic and pressure ulcers: a systematic review

**DOI:** 10.3389/fbioe.2026.1912135

**Published:** 2026-09-24

**Authors:** Lok Lam Lorraine Cheng, Chun Chit Cheung, Yiu Che Chan

**Affiliations:** Division of Vascular & Endovascular Surgery, Department of Surgery, Queen Mary Hospital, University of Hong Kong, Hong Kong, Hong Kong SAR, China

**Keywords:** 3D bioprinting, lower extremity ulcers, regenerative medicine, scaffold, ulcers

## Abstract

**Aim:**

Three-dimensional (3D) bioprinting enables fabrication of bioink-based scaffolds that mimic native tissue architecture. This systematic review aims to evaluate the safety profile and healing times of 3D-bioprinted scaffolds clinically tested in human lower limb ulcers.

**Methods:**

A systematic review was conducted in accordance with PRISMA guidelines. PubMed, Embase, Scopus, and Web of Science were searched from January 2000 to June 2025. Inclusion criteria encompassed only clinical studies; animal, *in vitro*, and non-clinical reports were excluded. Risk of bias was assessed using the revised Cochrane risk-of-bias tool for randomized trials (RoB 2) for randomized controlled trials and the Methodological Index for Non-Randomized Studies (MINORS) for non-randomized studies. Results were synthesized narratively due to clinical and methodological heterogeneity across studies.

**Results:**

Of 476 records screened, eight studies met the inclusion criteria: two randomized controlled trials and six single-arm studies. The combined sample size across all studies was 139 patients. Both RCTs reported a statistically significant reduction in time to complete epithelialization versus standard dressings. In the single-arm studies, >80% of ulcers achieved complete epithelialization within 12 weeks. No treatment-related serious adverse events were reported. Risk-of-bias assessments identified methodological limitations across studies (variable randomization and blinding, small sample sizes, and limited follow-up).

**Conclusion:**

Current clinical evidence indicates that 3D-bioprinted scaffolds, particularly Autologous Minimally Manipulated Homologous Adipose Tissue (AMHAT) and polylactic-acid gelatin constructs, represent a promising and apparently safe approach to accelerate ulcer healing and improve cosmetic outcomes. However, the number of patients included in each study were small with relatively short follow-up. Larger, high-quality randomized trials with longer follow-up are needed to confirm efficacy and long-term safety.

## 1 Introduction

Lower extremity ulcerations are a major global healthcare burden, affecting up to 49 million people worldwide every year with a cumulative lifetime risk of 1.0%–1.8% (Schneider et al., 2021). Standard care—debridement, offloading, compression, revascularization, and dressings—often yields suboptimal healing, with high complication and mortality risks. There is a clear need for new strategies that accelerate wound closure and enhance delivery of biomaterials, pharmaceuticals, and stem cells to the wound microenvironment (SALENIUS et al., 2021).

Tissue engineering was developed inthe 1980 s to generate implantable tissue-like constructs (Berthiaume et al., 2011). The scaffolds of these constructs are ideallybiocompatible; biodegradable without toxic by-products; mechanically suitable for the implantation site and surgical handling; highly porous with interconnected architecture; and amenable to scalable, cost-effective manufacturing (O’Brien, 2011; Tallapaneni et al., 2021).

3D bioprinting extends traditional scaffold fabrication by enabling precise spatial placement of biomaterials and cells within a tissue framework, allowing controlled pore size, porosity and geometry with enhanced repeatability (Bishop et al., 2017; Liu et al., 2022). In this paper 3D bioprinting is defined as an additive manufacturing technology that deposits bioinks in a layer-by-layer deposition, allowing it to yield constructs that more closely resemble native tissue (Antezana et al., 2022). Bioinks can be based on natural polymers (e.g., alginate, gelatin) or synthetic polymers (e.g., polycaprolactone, polyethylene glycol), and some techniques even enable direct cell printing (Gungor-Ozkerim et al., 2018). Applications relevant to ulcer management include adipose tissue grafting, skin substitutes, and antimicrobial hydrogels (Li et al., 2024).

This paper aims to address the research gap, specifically in clinical studies of 3D bioprinted scaffolds on lower limb ulcer healing, which includes diabetic, venous, arterial, and pressure ulcers. While these ulcers differ in their etiology, they share fundamental pathophysiology including dysregulation of inflammation, tissue hypoxia and extracellular matrix (ECM) degradation (Xiques-Molina et al., 2026). 3D bioprinted scaffolds mimic the ECM and provide structure integrity to the ulcer site to support cell viability and promote angiogenesis (Abuhamad et al., 2024). Thus, the action of bioprinted scaffolds on ulcers of different etiologies are comparable.

## 2 Methods

### 2.1 Data sources and search strategy

This systematic review was conducted and reported in accordance with the Preferred Reporting Items for Systematic Reviews and Meta-Analyses (PRISMA) guidelines. The search strategy, inclusion criteria, study selection and data analysis plan were defined prior to initiating the search and documented internally. No review protocol was prospectively registered on any public registry such as PROSPERO. A literature search was performed using PubMed, Scopus, Web of Science, and Embase on 12 June 2025, with the following search terms (‘Diabetic foot ulcer*' OR ‘Diabetic ulcer' OR “Diabetic foot” OR “foot ulcer OR “'Pressure ulcer*' OR ‘Venous ulcer*' OR ‘Arterial ulcer' OR ‘Arterial wound' OR ‘Venous wound' OR ‘diabetic wound*' OR ‘ulcer') AND (‘3D bioprint*' OR 3D bioprint *OR ‘bioprinting' OR ‘3D print*' OR ‘3D-print*' OR ‘3D-bio-print*' OR ‘3D cell printing' OR ‘3D scaffold' OR ‘3D prototyping' OR ‘Three-dimensional bioprint*' OR ‘Three dimensional printing' OR ‘bioink' OR ‘bio-ink'). The specific search syntax for each platform is in Supplementary Material 1. These search terms were aimed at maximizing the number of studies of various ulcer treatments related to 3D bioprinting. “Scaffolding” was considered but produced too many unrelated studies and was omitted from the search terms. We considered omitting “3D printing” to screen out papers unrelated to bioprinting, such as 3D printed insoles, but there were too little results under the other bioprint related search terms.

### 2.2 Study selection

Records identified through the search strategy were imported into Covidence, an online Systematic Review Tool. Each study underwent title and abstract screening by two independent reviewers. Duplicate studies were removed manually and automatically by Covidence. There were conflicts on whether small case studies should be included in the analysis and were ultimately accepted due to the small pool of studies. Inclusion of grey literature was discussed but only peer-reviewed studies were included in the final analysis to ensure the quality of the evidence. Full-text assessment of potentially eligible studies was then performed by both reviewers. Number of studies included in each step is shown in Figure 1. Manual reference screening was done and no other new studies were identified.

**FIGURE 1:**
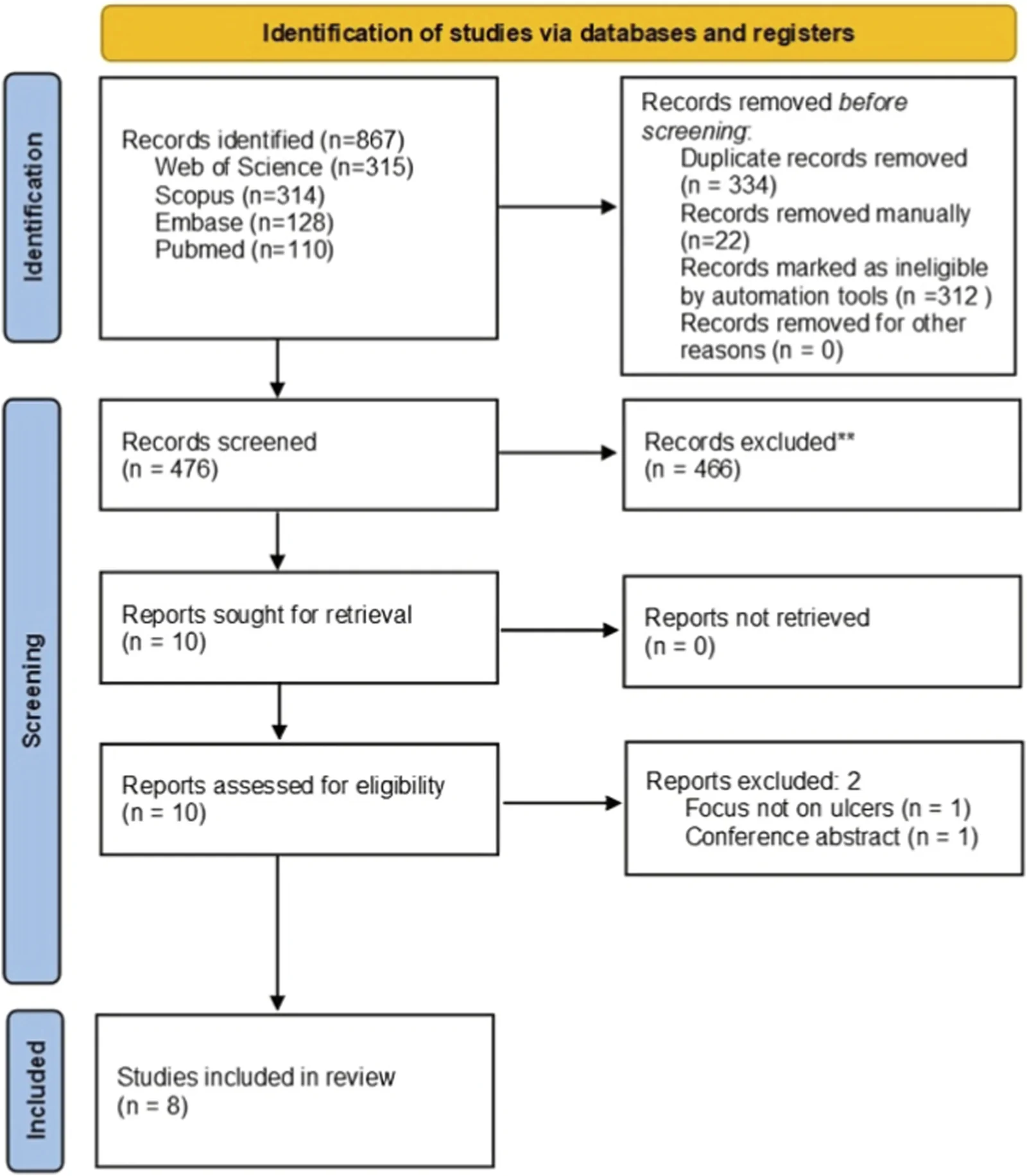
PRISMA flow diagram showing the process of study selection.

The primary objective of this review was to compare the effectiveness of 3D-bioprinted dressings with traditional dressings. Due to the lack of comparative studies, single-arm studies were included. Our population included studies with adult patients (age ≥18 years) with one or more lower extremity ulcers. For intervention, all types of ulcer dressing and scaffolding that involved 3D bioprinting technology in the production process were considered regardless of materials used. To be included, a study had to evaluate wound healing outcomes, such as time to complete wound healing or rate of wound size reduction.

The inclusion criteria adopted were studies published between January 2000 and June 2025, studies in all languages, original studies related to the use of 3D bioprinting technology in lower extremity ulcer management, limited to clinical trials and studies done on humans only, with reported results of ulcer recovery, all original clinical study designs including case reports and case series. A translator was to be employed if the study was written in a language the reviewers could not understand to broaden the scope of this study. The exclusion criteria were conference abstracts, systematic reviews, *in vitro* studies, animal *in vivo* studies, studies without 3D bioprinting technology and studies not focusing on lower limb ulcers.

### 2.3 Data extraction and synthesis

Data of the studies were extracted together by both reviewers. The results of each individual study was charted with their key findings to have a general overview of the effect 3D bioprinting technology has on boosting ulcer healing.

There were three main presentations of the outcome. Firstly, measurement of complete epithelialization within a time point. Secondly, measurement of mean wound size and rate of change. Thirdly, measurement to time of full closure of wound. Some studies only reported the percentage of total number of wounds completely epithelialized within a time point as an endpoint and this was accepted. Other outcomes extracted include wound duration, follow up duration, adverse effects, ease of use of the dressings and patient satisfaction. Due to the heterogeneity of the outcome measures, meta-analyses and plots were not feasible.

### 2.4 Quality assessment

Quality assessment for the two randomized controlled trials was conducted in accordance with the revised Cochrane risk-of-bias tool for randomized trials (RoB 2.0). Figure 2 shows the overall results in a visual manner addressing five domains. Two reviewers independently applied the tool to both studies and recorded justifications for judgements in each domain (low; high; some concerns). Discrepancies were resolved by discussion to reach a consensus. Following RoB 2.0 guidance, an overall summary judgement was given for each outcome and overall RoB was determined by the highest ROB level in any domain assessed.

**FIGURE 2:**
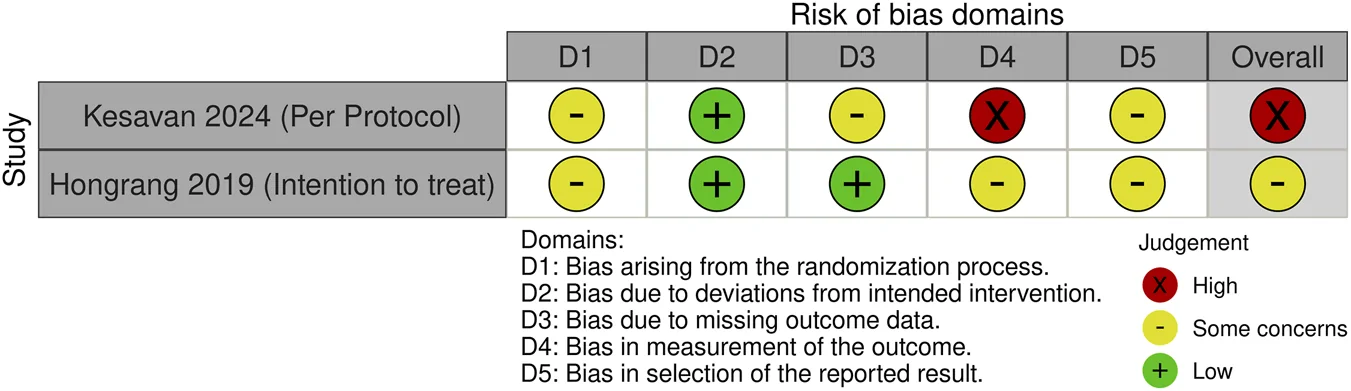
Cochrane risk-of-bias tool (RoB 2.0) assessment for RCTs.

As for the non-randomized trials, the gold standard ROBINS-I could not be conducted as all six studies were single arm studies and confounding cannot be addressed without a comparator and are at “critical risk of bias” in the RoB 2.0. The Methodological Index for Non-Randomized Studies (MINORS) was chosen as this tool was specifically developed for non-comparative studies (Slim et al., 2003). MINORS consists of eight criteria, and 0 marks are given if the information is not reported, one if it is reported but inadequate and two if it is adequate. Two independent reviewers assessed each paper and conflicts were discussed. The quality assessment of the included cohort studies is presented in Figure 3. Only the four cohort studies could be evaluated as there is no tool to evaluate single case studies.

**FIGURE 3:**
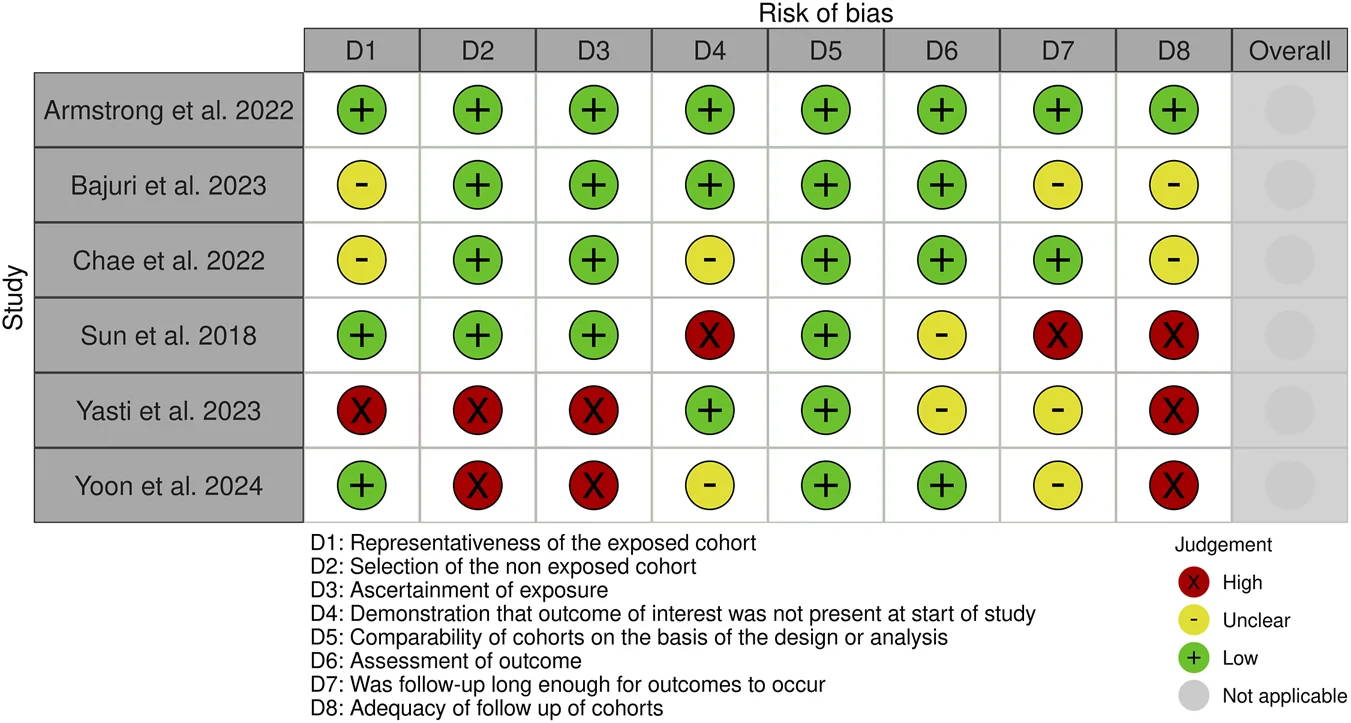
MINORS Risk of bias assessment for non-randomized studies.

### 2.5 Certainty assessment

Two reviewers independently assessed the certainty for prespecified outcomes using the GRADE approach. For randomized trials, certainty starts at high and is downgraded for risk of bias, imprecision, inconsistency, indirectness, and publication bias. Nonrandomized data, if used for context, would be considered with a GRADE-adapted approach. GRADE concepts and documentation were used to structure the judgments, and a Summary of Findings table was not used in this version. The assessment is in Supplementary Material 2.

## 3 Results

A total of 3,327 published articles on 3D bioprinting were identified in the Web of Science Core Collection between 2007 and 2022 (Ding et al., 2023). However, much of the wound-healing literature remained confined to *in vitro* or animal studies, with a 2025 scoping review reporting only 11 clinical bioprinting studies (Biondo et al., 2024; Briones et al., 2025). Extrusion-based systems were not able to reproduce the microscale architecture of native human tissues, and collagen was used in only about 15% of studies due to printing challenges (Holland, 2025). Across Web of Science, Scopus, EMBASE, and PubMed, 867 records were initially identified. After removal of 22 manually identified duplicates and 312 duplicates flagged by Covidence, 476 records remained for title and abstract screening, of which 466 were excluded as irrelevant. Ten full-text articles were assessed, and eight met the inclusion criteria. These comprised two RCTs evaluating a bionic polylactic acid/gelatin nanofiber membrane (Chen, 2019) and AMHAT (Kesavan et al., 2024) shown in Table 1, and six single-arm studies or case reports using AMHAT and 3D-printed scaffolds or powders shown in Table 2. Two studies were excluded: Kim (2022), a conference abstract reporting patients already included in five other publications, and Matsumura et al., 2025, which focused on skin cancer excision defects rather than chronic ulcers.

**TABLE 1:** Characteristics and results of the randomized controlled trials included in the study.

Author, year of publication and country	Number of test subjects and control subjects	Type of ulcer	Wound duration	Test group intervention	Control group	Dressing used	Follow up duration	Outcome measured	Results	Adverse effects	Risk of bias rating using RoB 2.0	Overall certainty of evidence (GRADE assessment)
Kesavan et al., 2024 (India)	Test group (n = 17) and control group (n = 16)	Neuropathic diabetic foot ulcers Size: 1 cm^2^–5 cm^2^	More than 8 weeks	3D-bioprinted autologous minimally manipulated homologous adipose tissue (3D-AMHAT) with fibrin gel scaffolding	Standard wound care	A silicone wound contact layer was used as primary and a foam dressing as the secondary to prevent pressure and infection	12 weeks	Complete epithelialization wound healing at 12 weeks	100% of test group subjects 50% of control subjects	None reported	High	Low
Chen, 2019 (China)	Test group (n = 30) and control group (n = 30)	Varicose vein ulcers Test: 9.11 ± 3.59 Control: 8.91 ± 3.72	More than 4 weeks	3D electrospun bionic polylactic acid/gelatin nanofiber membrane	Standard wound care	The wound was covered in petroleum jelly and an outer layer of sterile gauze with pressure bandaging and external fixation	28 days	Wound healing time	Test: 44.63 ± 12.37 Control: 54.10 ± 13.19	None reported	Some concerns	Low
								Dressing change frequency	Test: 6.73 ± 1.96 Control: 8.30 ± 2.07			
​	​	​	​	​	​	​	​	Wound healing rate at 3, 7, 14, 21 and 28 days (c: control, t: Test)	D3: 13.18 ± 1.90 (t) 10.92 ± 1.77 (c) D7: 42.69 ± 7.27 (t) 16.94 ± 6.75 (c) D14: 62.17 ± 10.54 (t) 31.04 ± 10.26 (c) D21: 73.91 ± 12.45 (t) 50.02 ± 14.63(c) D28: 83.12 ± 14.58 (t) 67.18 ± 17.61(c)	​	​	​
								Visual analogue scale	Test: from 5.67 ± 1.21 to 0.93 ± 0.74 Control: from 5.57 ± 1.33 to 3.17 ± 0.87			

**TABLE 2:** Characteristics and results of the non-randomized trials included in the study.

Author, year of publication and country	Type of study	Population	Type of ulcer and mean area	Wound duration	Type of 3D bioprinting used	Dressing used	Outcome measured	Results	Adverse effects	Risk of bias rating using MINORS	Overall certainty of evidence
Armstrong et al., 2022 (United States)	Single arm pilot study	10 patients	Diabetic foot ulcer Mean area 2.7 ± 2.43 cm^2^	More than 4 weeks	3D-bioprinted autologous minimally manipulated homologous adipose tissue (3D-AMHAT) with fibrin gel scaffolding	Primary nonadherent silicon dressing Secondary foam dressing followed by cast padding	Complete epithelialization within 12 weeks	6 of 10 wounds (60%) achieved complete epithelialization within 12 weeks	None reported	Low risk	Very low
							Mean time to heal	49.1 days			
Bajuri et al., 2023 (Malaysia)	Single arm pilot study	10 patients	Diabetic foot ulcer Mean size was 27.00 ± 43.99 cm^2^	Not mentioned	3D-bioprinted autologous minimally manipulated homologous adipose tissue (3D-AMHAT) with fibrin gel scaffolding	Primary silicon wound dressing Secondary foam wound dressing	Complete epithelialization within 12 weeks	7 of 10 wounds (70%) achieved complete epithelialization within 12 weeks	None reported	Moderate risk	Very low
							Wound size reduction	Significant (p < 0.05) progressive reduction			
							Time to healing	Slowest patient at 17 weeks			
Chae et al., 2022 (Korea)	Case study	67-year-old woman	Diabetic foot ulcer	Not mentioned	3D-bioprinted autologous minimally manipulated homologous adipose tissue (3D-AMHAT) with fibrin gel scaffolding	​	Complete epithelialization (96%)	34 days	None reported	Not applicable	Very low
Sun et al., 2018 (China)	Case series	1 patient	Pressure ulcer	2 years	Poly-L-lactic acid and gelatin 3D-printed scaffold membrane	Primary dressing: Petroleum-impregnated gauze Secondary dressing: absorbent dressing	Healing time	28 days	None reported	Moderate risk	Very low
		2 patients	Pressure ulcer	1 month; 6 months	Poly-L-lactic acid and gelatin 3D-printed scaffold powder			54 days			
		2 patients	1 pressure ulcer, 1 diabetic ulcer	2 years; >4 weeks	Poly-L-lactic acid and gelatin 3D-printing powder mixed with PRF			PU: 11 days Dfu: 14 days			
Yastı et al., 2023 (Turkey)	Single arm pilot study	20 patients	Diabetic foot ulcer Mean size 7.529 cm^2^	48.75 days	3D-bioprinted autologous minimally manipulated homologous adipose tissue (3D-AMHAT) with fibrin gel scaffolding	Primary dressing: negative pressure non-adherent dressing Secondary dressing: Saline-soaked cotton gauze	Complete epithelialization within 9 weeks	19 of 20 wounds (95%)	3 cases of mild subcutaneous bleeding, controlled by bandaging	Moderate risk	Very low
Yoon and Jin, 2024 (Korea)	Case study	78-year-old female patient with renal failure and severe peripheral arterial disease	Diabetic foot ulcer	Not mentioned	3D-bioprinted autologous minimally manipulated homologous adipose tissue (3D-AMHAT) with fibrin gel scaffolding	Primary dressing: nonadherent silicon dressing Secondary dressing: Foam dressing	Wound size reduction by 12 weeks	Lateral wound completely epithelized by 12 weeks, wound on heel more than 75% epithelized	None reported	Not applicable	Very low

It presents the author, year, country, study design, sample size, ulcer type, intervention, comparator or dressing used, outcomes measured, and results of two randomized controlled trials, with overall better results from the test groups using bioprinting than the control groups using standard wound care.

Table listing six studies on 3D bioprinting for ulcer treatment. The studies include single-arm pilot studies, case studies, and a case series from India, China, the United States, Malaysia, Korea, and Turkey. It shows participant numbers, ulcer type and size, intervention, dressing, outcomes, and results.

Together, these studies included 139 patients treated at multiple centers in six countries (Sun et al., 2018; Armstrong et al., 2022; Chae et al., 2022; Bajuri et al., 2023; Yastı et al., 2023; Yoon and Jin, 2024). Table 1 provides an overview. The primary endpoint in most reports was time to complete epithelialization within 12 weeks, with systematic documentation of wound healing.

RoB 2.0 was done on both RCTs overall showing moderate to high concerns with bias. All four cohort studies showed moderate quality when assessed with MINORS, scoring 11–13. Armstrong et al. (2022) performed better than the other two AMHAT studies due to their use of consecutive patients at the same center. Bajuri et al. (2023) had ambiguous inclusion criteria affecting the objectivity of the subject selection process and scored most poorly. Notably, no studies had blinded assessments of the study endpoints or prospective calculation of study size.

In the AMHAT studies, adipose tissue transfer was used to generate patient-specific extracellular matrix (ECM) constructs. Adipose tissue was harvested from the lower abdomen under local anesthesia by liposuction (Armstrong et al., 2022; Fanniel et al., 2025). The included studies employed the Dr. INVIVO AI Regen KIT (ROKIT Healthcare) to process the harvested tissue into nano-fat via a sequential filtration system, preserving the native regenerative characteristics of adipose tissue while isolating ECM for patch fabrication, creating an adipose tissue based bioink. In some studies, the ulcer was scanned using the AiD Regen automated 3D modeling system to generate a three-dimensional model of the defect. Based on this model, a scaffold composed of medical grade polycaprolactone (PCL) was printed, and the prepared adipose based ECM was printed in layers via an extruder onto the scaffold. Fibrin glue was then applied to stabilize the construct and form a cohesive ECM patch suitable for implantation (Matsumura et al., 2025).

In terms of healing outcomes, the studies suggest that 3D-bioprinted dressings were associated with superior recovery rates compared with conventional dressings. In the randomized trial of AMHAT dressings for diabetic foot ulcers, complete epithelialization at 12 weeks was achieved in 100% of ulcers in the intervention group versus 50% in the standard care group (Kesavan et al., 2024). Other reports suggested a particular benefit in long-standing ulcers, including a case series of wounds present for 6 months to 2 years, in which complete closure occurred within approximately 5 weeks of treatment (Sun et al., 2018). Chronic ulcers often harbor senescent fibroblast populations with reduced proliferative capacity and altered secretory profiles, contributing to persistent non-healing (Frykberg and Banks, 2015). The preliminary favorable results in elderly and comorbid patients suggest that 3D-bioprinted scaffolds may partially overcome these limitations.

Time to recovery accelerated in several studies compared to published benchmarks for standard care in diabetic foot ulcers (Yang et al., 2022). In the AMHAT randomized trial, all ulcers in the intervention arm achieved complete epithelialization by 12 weeks, and most showed near-complete healing by around 4 weeks, substantially faster than in controls (Kesavan et al., 2024). In the non-randomized trials, the results were generally good with some outliers. Yastı et al., 2023 reported complete healing in 95% of patients by the ninth week, while Chae et al. (2022) documented a diabetic wound reaching 96% epithelialization in under 5 weeks. Bajuri et al. (2023) reported complete epithelialization in 70% of wounds at 12 weeks, and (Yoon and Jin, 2024) described a patient in whom a lateral foot wound closed by 12 weeks while a heel wound remained 75% epithelialized. Armstrong et al. (2022) reported the least favorable results, with 40% of ulcers failing to close within 12 weeks. Interestingly, Bajuri et al. (2023) reported the largest mean wound size (27.00 ± 43.99 cm^2^), while Armstrong et al. (2022) documented smaller ulcers (mean 2.7 cm^2^, range 0.1–6.9 cm^2^) with depths ranging from 0.1 to 0.4 cm. However, Yastı et al., 2023 highlighted that even a superficially small ulcer (0.5 cm^2^) with a depth of 1.2 cm could behave as a complex wound, underscoring the importance of depth as a confounding factor (Yastı et al., 2023).

Patient- and clinician-reported outcomes were in favour of3D-bioprinted dressings in the studies included. In the randomized trial of a PLA/gelatin nanofiber membrane for varicose ulcers, pain scores during dressing changes decreased markedly in the experimental group (from 5.67 to 0.93 on a visual analog scale), compared with a reduction from 5.57 to 3.17 in controls, with no baseline difference between groups (Chen, 2019). Secondary tissue trauma was also reduced: average trauma scores were 1.30 ± 0.47 versus 1.93 ± 0.87 in the control group, indicating less adhesion and bleeding upon removal. The experimental group required fewer dressing changes (6.73 vs. 8.30 on average), reflecting both improved healing and reduced procedural burden. Clinicians rated ease of application significantly higher for the nanofiber membrane (mean score 1.27 vs. 3.23), and similar ease-of-use and comfort scores were reported in a PLLA/gelatin scaffold series, where most cases were rated “very easy” or “easy” by both clinicians and patients (Sun et al., 2018). These advantages likely reflected the membrane’s capacity to maintain a moist yet breathable wound environment, its high absorbency for excess exudate, and its ability to be removed or left in place without causing secondary trauma due to its biodegradable nature. The study suggests that traditional petroleum-based dressings often adhere firmly, leading to pain, bleeding, and additional tissue injury during dressing changes in comparison.

Clinicians using AMHAT-based 3D bioprinting further emphasized practical benefits: the technology provided structural support to fat grafts, allowing them to conform to irregular wound geometries without additional structural materials, and the relatively short operating time makes the technique attractive for patients at high surgical risk. Yoon and Jin, (2024) noted that these features expand therapeutic options for patients with multiple comorbidities who might otherwise be poor candidates for more extensive reconstructive procedures. A technique described by Sun et al. (2018) turning bioprinted scaffolding into powder and combining with platelet-rich fibrinogen to create a hydrogel can allow maximum adaptability with good adherence. Collectively, the available evidence, although limited in size and heterogeneity, suggests that 3D-bioprinted scaffolds may potentiallyoffer promising improvements in healing rates, healing time, and patient and clinician experience compared with conventional dressings.

## 4 Discussion

This is the first report in contemporary literature to solely focus on reviewing clinical studies on 3D bioprinted scaffolds on lower limb chronic ulcers. In the past 20 years, the average time for a diabetic foot ulcer to heal has remained at approximately 6 months (Sørensen et al., 2019). Our findings suggested that various methods of 3D bioprinted scaffolds decreased recovery time in comparison to conventional dressings and achieved complete healing by 12 weeks in most patients.

Previous studies on 3D bioprinting in ulcers either only concentrated on diabetic ulcers, or included mostly *in vitro* studies (Biondo et al., 2024; Li et al., 2024). This review of the literature aims to address a significant gap in clinical use of 3D bioprinted scaffolds, and we found that 3D bioprinting methods showed early promising decreases in the time to healing compared to standard methods, and this effect was seen broadly in vascular, diabetic and pressure ulcers. Overall, patients and clinicians were satisfied with these specific types of 3D bioprinted scaffolds in terms of ease of application and comfortability. Regarding adverse effects, only Yastı et al., 2023 mentioned three mild cases of subcutaneous bleeding, which suggest that these scaffolds have good safety profiles but should not be interpreted as definite evidence of long term safety due to the short duration of these studies.

This study also provides clinicians with an overview of emerging treatment options, particularly the two major directions in 3D bioprinted scaffolding–AMHAT and PLA/gelatin scaffolds. Notably, this study includesstudies from all languages, including a Chinese randomized control trial that was not included in any previous studies (Chen, 2019).

To better interpret these clinical outcomes, it is important to consider the structural design and biological functions of the scaffold types identified in this review. Autologous fat grafting for wound healing can stimulate collagen fiber synthesis, stimulate angiogenesis and promote thickening of the dermis and subcutaneous tissue through the action of various growth factors including vascular endothelial growth factor and insulin-like growth factor. (Mojallal et al., 2009). The 3D bioprinting technology and PCL/fibrin scaffolding allows AMHAT to maintain structure when placed on top of the wound. This gives AMHAT a theoretical advantage compared to traditional fat grafting in preventing fat necrosis due to sufficient vascular perfusion across the wound surface as well as the feasibility of direct postoperative monitoring.

PLA/gelatin scaffolding has good biocompatibility, and its final degradation products are CO2 and H2O which are non-toxic to the human body and have an additional benefit in providing hydration to the wound. PLA provides the tensile strength and elasticity (Chen et al., 2021) while gelatin is a collagen precursor that comprises the arginine-glycine-aspartic acid sequence that binds to cell surface integrins to stimulate proliferation and adhesion (Sonwane et al., 2025). Electrospinning provides an additional benefit as it can generate loosely connected, highly porous scaffolds which mimic extra-cellular matrix structure and promote cell infiltration and granulation tissue formation (Gao et al., 2021).

However, several factors may limit the generalizability of these findings. There is substantial clinical heterogeneity involved as the studies included a mix of ulcer types that have distinct underlying pathologies that influence their healing potential, as well as patients with different comorbidities. The small sample sizes hindered the ability to perform comparative analyses. The studies included have varied outcome measures, methodological uncertainties, and some concerns with bias. In addition, the utilization of platelet rich plasma and adipose tissue in the constructs affect the validity regarding the effectiveness of 3D bioprinting on its own.

As 3D bioprinting remains a relatively novel field, most of the studies are early positive pilot studies and may lead to publication bias. (Murad et al., 2018). Commercial bias must also be considered as a significant amount of the studies included in this paper were of the proprietary AMHAT platform and funded by its parent company. As for the limitations of this systematic review, the review protocol was not prospectively registered which may lead to methodological bias.

This review highlights the need for further research in some fields of 3D bioprinting. Firstly, many design factors may influence the outcome of treatment with bioprinted scaffolds, including nozzle size, bioink viscosity and the formation of crosslinks which should be reported in future clinical studies (Karvinen and Kellomäki, 2023). Regarding the broader use of 3D bioprinting, the Dr. INVIVO AI Regen Kit, which is a disposable kit with 3D bioprinting accessories and ECM bioink processing tools needed to create the AMHAT patch is estimated to cost 5000 USD (Armstrong et al., 2022). Further economic evaluations are needed to determine its cost effectiveness for ulcer management. Legislatory problems regarding 3D bioprinted scaffolds should also be studied. For example, the US Food and Drug Association (FDA) may choose to consider AMHAT under lighter regulatory burden similar to traditional fat grafting due to its minimally manipulated nature, but the FDA could also consider it a combination product, which would require additional tests and up to 10 years to get it approved (Perin et al., 2026). There are also concerns regarding standardizing an autologous bioink, and difficultes in maintaining industry standards during reproduction.

Beyond ulcer treatment, 3D bioprinting technologies demonstrate broader clinical potential. The AiD Regen technology used in some AMHAT studies is a novel method for wound measurement that can generate precise and efficient 3D wound models reducing inter-operator variability to produce a standardized measurement of wound area and volume (Chae et al., 2022). Apart from its use in treating ulcers, AMHAT has also demonstrated promising applications in other medical areas such as managing skin cancer and aiding healing post-amputation (Yoon and Jin, 2024; Matsumura et al., 2025).

Despite the growing interest in bioprinting technologies, a gap remains between laboratory research and clinical translation. A systematic review done in 2024 found 112 articles relevant to 3D bioprinting technology in the treatment of diabetic foot ulcers, yet only eight clinical trials were included in this review. (Li et al., 2024). This discrepancy highlights the need for more high-quality human studies to validate the clinical effectiveness and safety of 3D bioprinted scaffolds in ulcer management.

## Data Availability

The original contributions presented in the study are included in the article/Supplementary Material, further inquiries can be directed to the corresponding author.
