# Supplementary material for: Clinical trials on 3D bioprinted scaffolds for lower extremity vascular, diabetic and pressure ulcers: a systematic review
Source: Front Bioeng Biotechnol. 2026 Sep 24;14:1912135. doi: 10.3389/fbioe.2026.1912135 (PMC13645774; doi:10.3389/fbioe.2026.1912135)
Supplement: Supplementary file 1 [file Table1.docx]

| **AMHAT compared to Standard Care for lower limb ulcers - RCT**  **Bibliography:**  **Kesavan R, Sheela Sasikumar C, Narayanamurthy VB, Rajagopalan A, Kim J. Management of Diabetic Foot Ulcer with MA–ECM (Minimally Manipulated Autologous Extracellular Matrix) Using 3D Bioprinting Technology – An Innovative Approach. Int J Low Extrem Wounds. 2024;23(1):161-168. doi:10.1177/15347346211045625** | | | | | | | | | | | |
| --- | --- | --- | --- | --- | --- | --- | --- | --- | --- | --- | --- |
| **Certainty assessment** | | | | | | | **Summary of findings** | | | | |
| **Participants (studies) Follow-up** | **Risk of bias** | **Inconsistency** | **Indirectness** | **Imprecision** | **Publication bias** | **Overall certainty of evidence** | **Study event rates (%)** | | **Relative effect (95% CI)** | **Anticipated absolute effects** | |
|  |  |  |  |  |  |  | **With Standard Care** | **With AMHAT** |  | **Risk with Standard Care** | **Risk difference with AMHAT** |
| **Complete epithelialization by 12 weeks** | | | | | | | | | | | |
| 33 (1 RCT) | serious | not serious | not serious | serious | none | ⨁⨁◯◯ Low | 16 | 17 | - | 16 | **100 % higher** (1.2 higher to 3.3 higher) |

**CI:** confidence interval

| **AMHAT compared to Standard Care for lower limb ulcers - Single arm**  **Bibliography:**  **Armstrong DG, Harris SG, Rasor Z, et al. Autologous Minimally Manipulated Homologous Adipose Tissue (AMHAT) for Treatment of Nonhealing Diabetic Foot Ulcers. Plast Reconstr Surg - Glob Open. 2022;10(10):e4588. doi:10.1097/GOX.0000000000004588**  **Bajuri MY, Kim J, Yu Y, Shahul Hameed MS. New Paradigm in Diabetic Foot Ulcer Grafting Techniques Using 3D-Bioprinted Autologous Minimally Manipulated Homologous Adipose Tissue (3D-AMHAT) with Fibrin Gel Acting as a Biodegradable Scaffold. Gels. 2023;9(1). doi:10.3390/gels9010066**  **Yoon HMS Woo Jin. Using 3D Bioprinted Autologous Minimally Manipulated Homologous Adipose Tissue for Limb Salvage in Treating Diabetic Foot Ulcer. Arch Plast Surg. 2024;51(03):332-336. doi:10.1055/a-2263-7957**  **Yastı A, Akgun A, Surel A, Kim J, Akın M. Graft of 3D Bioprinted Autologous Minimally Manipulated Homologous Adipose Tissue for the Treatment of Diabetic Foot Ulcer. Wounds Compend Clin Res Pract. 2023;35(1):e22-e28. doi:10.25270/wnds/21136**  **H. J. Chae, S. Lee, H. Son, S. Han, T. Lim. Generating 3D Bio-Printable Patches Using Wound Segmentation and Reconstruction to Treat Diabetic Foot Ulcers. In: 2022 IEEE/CVF Conference on Computer Vision and Pattern Recognition (CVPR). 2022:2529-2539. doi:10.1109/CVPR52688.2022.00257** | | | | | | | | | | | |
| --- | --- | --- | --- | --- | --- | --- | --- | --- | --- | --- | --- |
| **Certainty assessment** | | | | | | | **Summary of findings** | | | | |
| **Participants (studies) Follow-up** | **Risk of bias** | **Inconsistency** | **Indirectness** | **Imprecision** | **Publication bias** | **Overall certainty of evidence** | **Study event rates (%)** | | **Relative effect (95% CI)** | **Anticipated absolute effects** | |
|  |  |  |  |  |  |  | **With Standard Care** | **With AMHAT** |  | **Risk with Standard Care** | **Risk difference with AMHAT** |
| **Complete epithelialization at 12 weeks** | | | | | | | | | | | |
| 42 (5 non-randomised studies) | very serious | serious | not serious | very serious | none | ⨁◯◯◯ Very low | 0/0 | 33/42 (78.6%) | not estimable |  |  |

**CI:** confidence interval

| **PLLA and gelatin compared to Standard care for lower limb ulcers - RCT**  **Bibliography:**  **Chen Hongrang. Bionic polylactic acid/gelatin nanofiber membrane for repairing varicose ulcers in the lower extremities. Chin J Tissue Eng Res. 2019;23(26):4217-4224.** | | | | | | | | | | | |
| --- | --- | --- | --- | --- | --- | --- | --- | --- | --- | --- | --- |
| **Certainty assessment** | | | | | | | **Summary of findings** | | | | |
| **Participants (studies) Follow-up** | **Risk of bias** | **Inconsistency** | **Indirectness** | **Imprecision** | **Publication bias** | **Overall certainty of evidence** | **Study event rates (%)** | | **Relative effect (95% CI)** | **Anticipated absolute effects** | |
|  |  |  |  |  |  |  | **With Standard care** | **With PLLA and gelatin** |  | **Risk with Standard care** | **Risk difference with PLLA and gelatin** |
| **Complete epithelialization at 12 weeks (Recovery rate)** | | | | | | | | | | | |
| 60 (1 RCT) | serious | not serious | serious | not serious | none | ⨁⨁◯◯ Low | 30 | 30 | - | 30 | MD **9.5 days faster** (3 to 15.9 days faster) |
| **Pain reduction** | | | | | | | | | | | |
| 60 (1 RCT) | serious | not serious | serious | not serious | none | ⨁⨁◯◯ Low | 30 | 30 | - | 30 | MD **2.24 points lower** (0 to 0 ) |

**CI:** confidence interval; **MD:** mean difference

| **PLLA and gelatin compared to Standard Care for lower limb ulcers - Single arm**  **Bibliography:**  **Sun H, Lv H, Qiu F, et al. Clinical application of a 3D-printed scaffold in chronic wound treatment: a case series. J Wound Care. 2018;27(5):262-271. doi:10.12968/jowc.2018.27.5.262** | | | | | | | | | | | |
| --- | --- | --- | --- | --- | --- | --- | --- | --- | --- | --- | --- |
| **Certainty assessment** | | | | | | | **Summary of findings** | | | | |
| **Participants (studies) Follow-up** | **Risk of bias** | **Inconsistency** | **Indirectness** | **Imprecision** | **Publication bias** | **Overall certainty of evidence** | **Study event rates (%)** | | **Relative effect (95% CI)** | **Anticipated absolute effects** | |
|  |  |  |  |  |  |  | **With Standard Care** | **With PLLA and gelatin** |  | **Risk with Standard Care** | **Risk difference with PLLA and gelatin** |
| **Complete epithelialization at 12 weeks** | | | | | | | | | | | |
| 0 (1 non-randomised study) | very serious | not serious | serious | very serious | none | ⨁◯◯◯ Very low | 0 | 5/5  (100%) | - | - | **0**  (0 to 0 ) |

**CI:** confidence interval
