# Supplementary material for: Clinical trials on 3D bioprinted scaffolds for lower extremity vascular, diabetic and pressure ulcers: a systematic review
Source: Front Bioeng Biotechnol. 2026 Sep 24;14:1912135. doi: 10.3389/fbioe.2026.1912135 (PMC13645774; doi:10.3389/fbioe.2026.1912135)
Supplement: Supplementary file 2 [file Table2.docx]

Supplementary file 1

1. PubMed

Search Date: 12th June 2025

Filters Applied: Publication date from 2000/01/01 to 2025/06/12; Humans

Syntax:

(("Diabetic foot ulcer*"[tw] OR "Diabetic ulcer"[tw] OR "Diabetic foot"[tw] OR "foot ulcer"[tw] OR "Pressure ulcer*"[tw] OR "Venous ulcer*"[tw] OR "Arterial ulcer"[tw] OR "Arterial wound"[tw] OR "Venous wound"[tw] OR "diabetic wound*"[tw] OR "ulcer"[tw]) AND ("3D bioprint*"[tw] OR "bioprinting"[tw] OR "3D print*"[tw] OR "3D-print*"[tw] OR "3D-bio-print*"[tw] OR "3D cell printing"[tw] OR "3D scaffold"[tw] OR "3D prototyping"[tw] OR "Three-dimensional bioprint*"[tw] OR "Three dimensional printing"[tw] OR "bioink"[tw] OR "bio-ink"[tw])) AND (2000/01/01:2025/06/12[dp]) AND "humans"[Mesh Terms]

2. Embase (Elsevier)

Search Date: 12th June 2025

Filters Applied: Publication year 2000–2025; Human

Syntax:

(('Diabetic foot ulcer*':ti,ab,kw OR 'Diabetic ulcer':ti,ab,kw OR 'Diabetic foot':ti,ab,kw OR 'foot ulcer':ti,ab,kw OR 'Pressure ulcer*':ti,ab,kw OR 'Venous ulcer*':ti,ab,kw OR 'Arterial ulcer':ti,ab,kw OR 'Arterial wound':ti,ab,kw OR 'Venous wound':ti,ab,kw OR 'diabetic wound*':ti,ab,kw OR 'ulcer':ti,ab,kw) AND ('3D bioprint*':ti,ab,kw OR 'bioprinting':ti,ab,kw OR '3D print*':ti,ab,kw OR '3D-print*':ti,ab,kw OR '3D-bio-print*':ti,ab,kw OR '3D cell printing':ti,ab,kw OR '3D scaffold':ti,ab,kw OR '3D prototyping':ti,ab,kw OR 'Three-dimensional bioprint*':ti,ab,kw OR 'Three dimensional printing':ti,ab,kw OR 'bioink':ti,ab,kw OR 'bio-ink':ti,ab,kw)) AND [2000-2025]/py AND 'human'/de

3. Scopus

Search Date: 12th June 2025

Filters Applied: Publication year 2000–2025

Syntax:

(TITLE-ABS-KEY("Diabetic foot ulcer*" OR "Diabetic ulcer" OR "Diabetic foot" OR "foot ulcer" OR "Pressure ulcer*" OR "Venous ulcer*" OR "Arterial ulcer" OR "Arterial wound" OR "Venous wound" OR "diabetic wound*" OR "ulcer") AND TITLE-ABS-KEY("3D bioprint*" OR "bioprinting" OR "3D print*" OR "3D-print*" OR "3D-bio-print*" OR "3D cell printing" OR "3D scaffold" OR "3D prototyping" OR "Three-dimensional bioprint*" OR "Three dimensional printing" OR "bioink" OR "bio-ink")) AND PUBYEAR > 1999 AND PUBYEAR < 2026

4. Web of Science (Core Collection)

Search Date: 12th June 2025

Filters Applied: Custom Year Range (2000-01-01 to 2025-06-12)

Syntax:

TS=(("Diabetic foot ulcer*" OR "Diabetic ulcer" OR "Diabetic foot" OR "foot ulcer" OR "Pressure ulcer*" OR "Venous ulcer*" OR "Arterial ulcer" OR "Arterial wound" OR "Venous wound" OR "diabetic wound*" OR "ulcer") AND ("3D bioprint*" OR "bioprinting" OR "3D print*" OR "3D-print*" OR "3D-bio-print*" OR "3D cell printing" OR "3D scaffold" OR "3D prototyping" OR "Three-dimensional bioprint*" OR "Three dimensional printing" OR "bioink" OR "bio-ink"))
